# Book Reviews

**Published:** 1916-06

**Authors:** 


### BOOK REVIEWS

**Books mentioned may be inspected at and ordered through this office. So far as possible, books received in any month will be reviewed in the issue of the second month following.**

**Surgery in War,** Alfred J. Hull, F. R. C. S., Preface by Sir Alfred Keogh, K. C. B., M. D., 26 plates, 55 text figures, 390 pages, $4. P. Blakiston's Son & Co., Philadelphia.

This is a compact work, prepared by 8 collaborators of note, taking general principles and technic for granted and presenting succinctly, the special features of military surgery emphasized by the present war. Partly on account of marked differences in military methods, especially the terrible efficiency of modern missiles, and largely on account of the comparatively recent development of the X-ray, of bacteriologic technic and of a realization by military powers of the necessity of securing as high a degree of efficiency in medicine and surgery as in other branches of military work, the present war has introduced more new experience than any other and has required a decided modification of surgical technic and administration. For these reasons, this book should appeal not only to those anticipating the possibility of military practice or directly interested in military surgery, but the profession generally.

**The Ductless Glandular Disease,** Wilhelm Falta, Vienna, translated and edited by Milton K. Meyers, M. D., Philadelphia. Foreword by Archibald E. Garrod, M. D., F. R. C. P., F. R. S., London. Published by P. Blakiston's Son & Co., Philadelphia. Second edition, 101 illustrations, 673 pages; $7.

Nearly 70 pages are devoted to a classified bibliography. Among the subjects that might not be thought of as germane to such a work are eunuchism, hermaphroditism, castration, early and late, adipositas dolorosa, diabetes and some others. It is scarcely necessary to state that the work is not merely a pathologic or clinical treatise on a group of diseases. The ductless glands are considered in their various analogies and their conditions reduced to general principles so far as possible, but without going to the extreme of trying to make facts or limitations of knowledge fit preconceived theories. Heredity, metabolism, physiologic interrelations, are discussed. Hormones are given very brief attention, as the author does not think that the time is ripe to attempt a practical application of the hormone theory to the diseases under discussion.

---

**Transactions of the American Gastro-Enterological Assn. 1915** (18th annual session at Baltimore, May 10, 1915), 248 pages, illustrated, published by the Assn.

While essentially a collection of papers already published in medical journals after presentation to the Assn., the type has been entirely reset so that the volume is uniform in appearance. Lists of officers and members, constitution and by-laws are included. The papers adhere closely to gastro-enterology but by no means in the medical sense.

---

**Johns Hopkins Hospital Reports,** Vol. 17, 1916. 586 pages, illustrated.

This large volume includes only eight articles, all of great pathologic interest. Free Thrombi and Ball Thrombi in the Heart, J. H. Hewitt; Benzol as a leucotoxin, Lawrence Selling; Primary Carcinoma of the Liver, M. C. Winternitz; Statistic Experience Data of the Hospital, F. L. Hoffman; Origin and Development of the Lymphatic System, Florence R. Sabin; Nuclei Tuberis Laterales and the so-called Ganglion Opticum Basale, E. F. Malone; Venous Thrombosis during Myocardial Insufficiency, F. J. Sladen and M. C. Winternitz; Leukaemia of the Fowl, H. C. Schmeisser.

